# Driving better and safer HER2-specific CARs for cancer therapy

**DOI:** 10.18632/oncotarget.17528

**Published:** 2017-04-29

**Authors:** Xianqiang Liu, Nan Zhang, Huan Shi

**Affiliations:** ^1^ Department of Breast and Thyroid Surgery, Jinan Central Hospital Affiliated to Shandong University, Jinan, Shandong 250013, P.R. China; ^2^ Department of Oncology, Jinan Central Hospital Affiliated to Shandong University, Jinan, Shandong 250013, P.R. China; ^3^ Department of Oncology, Shandong Cancer Hospital Affiliated to Shandong University, Shandong Academy of Medical Sciences, Jinan, Shandong, P.R. China

**Keywords:** chimeric antigen receptor, HER2, cancer, immunotherapy, toxicity

## Abstract

Given the clinical efficacy of chimeric antigen receptor (CAR)-based therapy in hematological malignancies, CAR T-cell therapy for a number of solid tumors has been actively investigated. Human epidermal growth factor receptor 2 (HER2) is a well-established therapeutic target in breast, as well as other types of cancer. However, HER2 CAR T cells pose a risk of lethal toxicity including cytokine release syndrome from “on-target, off-tumor” recognition of HER2. In this review, we summarize the development of conventional HER2 CAR technology, the alternative selection of CAR hosts, the novel HER2 CAR designs, clinical studies and toxicity. Furthermore, we also discuss the main strategies for improving the safety of HER2 CAR-based cancer therapies.

## INTRODUCTION

The identification of human tumor-associated antigens (TAAs) recognized by the immune system initiated the field of cancer immunotherapy. Currently, various TAA-specific monoclonal antibody (mAb)-based immunotherapeutic strategies have been designed to redirect immune cell effector functions against tumors. Strategies based on chimeric antigen receptor (CAR) engineered T cells are emerging as the dominant approaches for adoptive cellular therapy (ACT) [1, 2].

Recent clinical trials by several centers have demonstrated highly compelling efficacy in patients with CD19-expressing hematological malignancies [3–6]. However, comparable therapeutic activity of CARs has not been achieved in solid tumors [7]. A rapidly growing number of clinical trials of CAR therapy have focused on solid tumors, targeting TAAs including carbonic anhydrase IX (CAIX) [8], folate receptor α (FRα) [9], L1 cell adhesion molecule (L1CAM) [10], mesothelin [11], carcinoembryonic antigen (CEA) [12], diganglioside GD2 [13], interleukin 13 receptor α2 (IL13Rα2) [14], EGFRVIII [15], human epidermal growth factor receptor 2 (HER2) [16], and fibroblast activation protein (FAP) [17]. HER2 is a well-established therapeutic target, and overexpression occurs in various solid tumors, such as breast, gastric, ovary, colon, bladder, lung, uterine cervix, head and neck, and esophageal cancer [18–21]. The key role of HER2 in the HER family signaling network led to the development of anti-HER2 mAbs, including trastuzumab and pertuzumab for cancer therapy [22]. T cells have better access to the tumor than antibodies and retain longer time *in vivo*. Therefore, CAR T-cell therapies targeting the HER2 receptor were actively evaluated in various types of cancers in preclinical and clinical settings.

Here, we review the development of the conventional HER2 CAR technology, novel designs, preclinical and clinical studies, toxicity and potential strategies for reducing toxicity of HER2 CAR therapy in an effort to improve the efficacy and safety of HER2 CAR therapy in solid tumors.

### Conventional HER2-specific car

**Figure 1:**
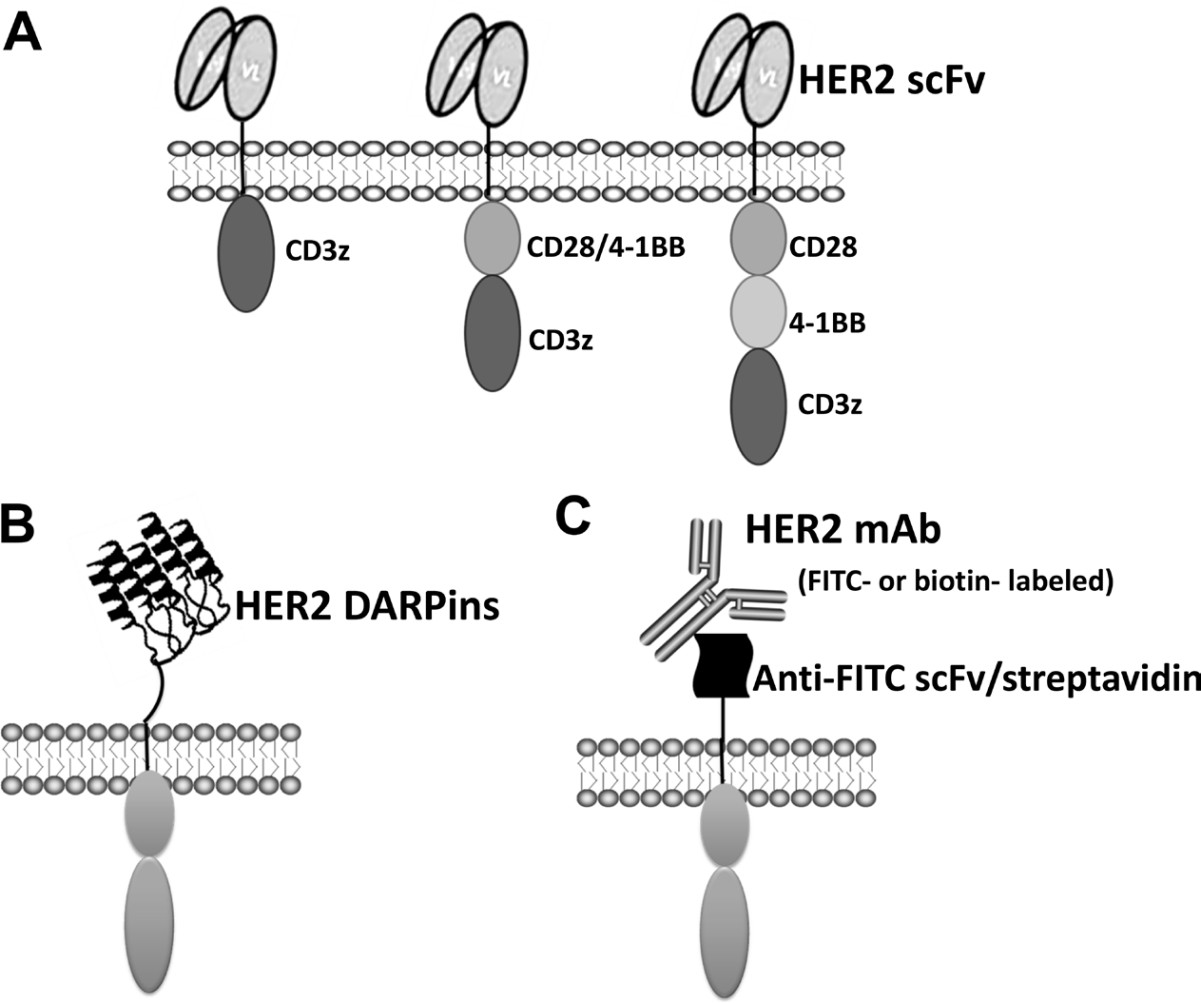
Different types of HER2-specific CAR design (**A**) First, second and third generation conventional CAR schemes containing scFv are shown. (**B**) Anti-HER2 DARPins-based novel second generation HER2 CAR. (**C**) HER2-specific antibody coupled second generation CAR.

Conventional CARs consist of an extracellular antigen recognition domain, commonly derived from a single chain variable fragment (scFv), a transmembrane (TM) domain, and signaling components. Incorporation of a flexible hinge between scFv and TM domain is able to improve CAR T cells expansion [23]. CARs are grouped into three generations [24–26] with one (1st generation), two (2nd generation), or three (3rd generation) signaling domains derived from CD3z and co-stimulatory molecules (Figure 1A). CARs can be transduced ex vivo using transfection, lentiviral or gamma retroviral vectors, or a transposon system [27–29]. CARs endow effector cell antigen-specific recognition, activation, and proliferation in an MHC-independent manner [24, 30].

### HER2-specific CAR T cells

T cells are found in the tumor microenvironment in various tumors and mediate tumor immune surveillance. T cells can also mediate long-lived, antigen-specific, effector and memory immune responses. A rapid approach for generating HER2 specific T cells is to engineer T cells to express a CAR. The history of developing HER2 CAR strategies is quite substantial. The first HER2 CAR was constructed and reported by Dr. Eshhar group in 1993 [31]. This study demonstrated the feasibility of construction of the 1st generation HER2 CAR containing either the zeta (ζ) chain of the TCR/CD3 complex or the gamma (γ) chain of the immunoglobulin receptor FcεRI. These CAR T cells specifically lyse HER2-postive target cells and produce interleukin-2 (IL-2), indicating the activation of CAR T cells. Another study presented a fully human HER2-specific scFv based 1st generation HER2 CAR [32], which has the potential to reduce the transgene immunogenicity that may hamper the success of CAR T cell trials that utilize scFvs of mouse origin. In one study [33], direct local injection of the 1st generation of HER2 CAR T cells into medulloblastomas in tumor-bearing mice resulted in the regression in all animals without exogenous cytokines support. However, significant recurrence was observed in all animals treated with these CAR T cells. In another study [34], 1st generation HER2 CAR T cells were able to eliminate 3-day pulmonary micrometastases. However, exogenous administration of IL-2 and a 5 to 8-fold increased dose of these CAR T cells were required to mediate regression of advanced, well-established 8-day macrometastases. These results suggested that first generation HER2 CAR T cells may be suboptimal for persistence and anti-tumor activity because T cells require two signals to become fully activated, and co-stimulation is crucial to the activation, proliferation and efficient immune response of CAR T cells.

HER2 has been targeted with 2nd and 3rd generation CAR T cells in breast cancer [35, 36], gastric cancer [37], glioblastoma [38], sarcoma [39], ovarian cancer [40], and osteosarcoma [41].The majority of studies have clearly demonstrated that the incorporation of co-stimulation molecules, such as CD28, 4-1BB, OX-40, ICOS, or CD27 [30, 36, 42–44] into CAR constructs offers better therapeutic benefit in preclinical and clinical settings. In one of these studies, Zhao et al. [36] found decreased transgene expression and increased apoptotic/dead cells of Herceptin-based 4D5 HER2 CARs with or without CD28 costimulation moiety. However, incorporation of 4-1BB signaling to 4D5 HER2 CARs increased expression of anti-apoptotic protein, greatly reduced the antigen-induced cell death (AICD) of the CAR T cells. Further investigation suggested that CAR-associated transgene decrease and cell death was due to apoptotic signaling transmitted from ITAMs within CD3z stimulated by a low level of HER2 expression in PBMCs. In addition, recent clinical studies [5, 45] have demonstrated that 4-1BB but not CD28 costimulatory domain significantly enhanced persistence of CD19 CARs T cells in patients. Another study [46] demonstrated that the 4-1BB endodomain can ameliorate key aspects of exhaustion induced by antigen-independent signaling or induced by persistent exposure to antigen. A more recent study [47] suggested one possible reason for the differential persistence was that 4-1BB CAR show an enhanced catabolic activity including fatty-acid oxidation and an enhanced mitochondrial biogenesis, which support central memory cell differentiation and prolonged persistence. While the CD28 CARs enhance glycolysis and yield more short-lived effector memory cell maturation. The choice of a CAR signaling domain can impact the subsequent fate of the T cells and the incorporation of 4-1BB in HER2 CARs may be superior to CD28 for persistence and antitumor activities. Therefore, 4-1BB based HER2 CAR T cells could be suitable for clinical applications.

### HER2-specific CAR CTLs

To achieve robust antitumor effects, CAR T cells must numerically expand and persist *in vivo*. However, CAR T cells may be suppressed by tumors that commonly express inhibitory ligands, such as programmed death-ligand 1 and 2 (PD-L1and PD-L2) [29, 48]. An alternative strategy to potentially increase the proliferation and long term activity of CAR T cells *in vivo* involves using T cells specific for antigens associated with chronic viral infection as the host for CAR manufacturing. For example, CAR engineered Epstein-Barr virus (EBV) or cytomegalovirus (CMV)-specific cytotoxic T lymphocytes (CTLs) can recognize both tumor targets and virus-infected cells through their chimeric and native receptors, and may survive longer *in vivo* than T cells without virus specificity.

Nakazawa et al. [49] successfully generated EBV CTLs expressing a HER2-CAR using the nonviral piggyBac-transposon system. *In vitro*, HER2-CAR EBV-CTLs could survive for over 100 days with cytokine support but in the absence of antigen stimulation and for a longer time in the presence of HER2 positive tumor or EBV-infected cells. Additionally, the long term of CAR expression is likely to provide distinct advantages for outstanding tumor control. *In vivo*, HER2-CAR EBV-CTL treatment induced tumor regression and resulted in a significant extension of survival time of tumor-bearing mice in a HER2 positive sarcoma xenograft model.

Similar HER2-CAR CMV-CTLs were successfully generated and evaluated in HER2 positive GBM patients. Sixteen HER2+ GBM patients with CMV−seropositive and radiological evidence of progression were enrolled in a Phase 1 clinical trial [50]. Infusions of HER2 CAR CMV CTLs were well tolerated without systemic toxicity and could persist for up to 3 months in the peripheral blood, based on a CAR specific amplicon using real-time PCR technology. A durable clinical benefit from treatment was observed in ∼38% of patients and the median survival was 11.6 months from the infusion. Thus, systemically administered HER2-CAR CMV-CTLs are safe and demonstrate potential therapeutic benefit. Collectively, virus-specific CTLs seem to offer distinct advantages as tumor-directed effector cells for CARs.

### HER2-specific CAR NK cells

Natural killer (NK) cells are currently gaining interest for cellular immunotherapy [51, 52]. NK cells are potent innate effector cells and form the first line of defense against diseases, including cancers. NK cells and cell lines (such as NK-92) do not require HLA matching and provide alternatives to the use of T cells, and importantly, can be used as allogeneic effector cells [53, 54]. Owing to the limited lifespan of NK cells, allogeneic and even autologous NK cells are expected to induce an immune response and should disappear relatively rapidly from the circulation; therefore, suicide genes are not required for CAR constructs. Compared to the cytokine release syndrome (CRS) of CAR T cells [55], CAR NK cells produce a different spectrum of cytokines including interferon-g (IFN-γ), granulocyte macrophage colony stimulating factor (GM-CSF) and interleukin-3 (IL-3), which cause limited damage to healthy tissues [56, 57].

The optimization of viral transduction and electroporation approaches has resulted in higher transduction efficiencies of primary human NK cells and NK cell lines [58–60]. Early clinical studies [61, 62] demonstrated the safety of infusions of NK-92 cells in cancer patients. Schönfeld et al. [63] generated a stable NK92 cell line expressing a humanized FRP5 HER2 CAR containing CD28 and CD3ζ signaling domains. These CAR NK cells efficiently lysed HER2-expressing tumor cells and exhibited serial targeted cell killing *in vitro*. *In vivo*, CAR NK cells retain target cell specificity and are capable of penetrating tissues and reaching distant tumor sites in orthotopic breast cancer xenografts. In addition, Zhang et al. [64] showed the same HER2 CAR expressing NK-92 cells lysed all HER2-positive established cell lines and primary GBM cells. In mice for GBM xenografts tumor model, these HER2 CAR engineered NK-92 cells treatment improved symptom-free survival upon repeated stereotactic injection compared with parental NK-92 cells.

However, because NK-92 cells are transformed and require irradiation prior to infusion to avoid the tumorigenic risk *in vivo*, they are not ideal for adoptive immunotherapy and do not reflect the biology of primary NK cells expressing a CAR [53]. In contrast, primary NK cells may have the potential of long-term persistence and proliferation in patients, a prerequisite for successful ACT. Kruschinskia et al. [65] showed that primary human NK cells can be efficiently transduced to express a HER2 CAR using the spinoculation protocol. They demonstrated that HER2 CAR NK cells respond specifically to all HER2-expressing targets including cell lines which were resistant to trastuzumab treatment. *In vivo*, HER2 CAR NK cell treatment did prevent tumor outgrowth in all mice, likely because CAR NK cells may overcome mechanisms of intrinsic inhibition, which limited their function in most of the animals that received mock-transduced NK cells. These data demonstrate that CAR-expressing NK cells represent a complementary and expandable therapeutic option to CAR T cells.

### HER2-specific CAR CIK cells and γδ T cells

Cytokine-induced killer (CIK) cells are MHC-unrestricted CD3^+^CD56^+^ cytotoxic lymphocytes that can be generated *in vitro* from PBMCs and cultured with the addition of IFN-γ, IL-2 and CD3 antibody (clone OKT3). CIK cells can be modified to express an antigen-specific CAR to enhance specific cytotoxicity of cancers. Yoon et al. [66] showed RNA encoding HER2 CAR electroporated CIK cells produce cytokines including IFN-γ, tumor necrosis factor-alpha (TNF-α), and granulocyte-macrophage colony-stimulating factor (GM-CSF), and show specific cytotoxicity against tumor cell lines expressing HER2. Treatment with HER2 CAR CIK cells led to significant inhibition of tumor growth *in vivo* compared with transfer of mock-transduced CIK cells, suggesting the potential therapeutic value of CAR CIK cells for cancers.

In the peripheral blood, the majority of T cells are αβ T cells, while γδ T cells contribute to only 5% of total CD3+ cells [67]. γδ T cells mediate anticancer immunity and γδ TCRs recognize cancer-associate antigens in a MHC-independent manner. Vγ9Vδ2 T cells, a major subset of γδ T cells that can be expanded by stimulation with bisphosphonate drug, such as Zoledronic acid have been tested in clinical trials for cancer therapy. Recently, Du et al. [68] described a K562 based aAPC method for co-expansion of CIK cells and Vγ9Vδ2 T cells, named as CIKZ cells. Importantly, HER2 CAR-modified CIKZ cells exhibited comparable killing efficacy to CAR-modified αβ T cells. The possibility of using CIKZ cells as an alternative cell source for CAR cell therapy warrants further evaluation in preclinical and clinical settings.

### Novel HER2-specifc CAR designs

The majority of HER2-specific CARs utilize a scFv, derived from anti-HER2 mAb, to enable antigen recognition. However, the antigen recognition domain of CARs is not confined to using scFv and other receptors and proteins have been utilized [69–72]. For example, Hammill et al. [69] demonstrated the feasibility of using designed ankyrin repeat proteins (DARPins) as alternative HER2-binding domains (Figure 1B). DARPins are novel binding molecules composed of ankyrin repeats (ARs), which stack together to function as protein binders [73, 74]. Each AR consists of 33 amino acids, which form into a β-turn followed by two anti-parallel α-helices and a loop reaching the β-turn of the next repeat. This study demonstrated that HER2 CARs with DARPins are as efficacious as conventional CAR with scFv. Thus, DARPins represent an attractive alternative to scFv and this study supports the further investigation of DARP-based CARs.

Another novel design is to engineer T cells to express a chimeric receptor that can directly bind to tumor-specific mAbs. When these engineered T-cells are transferred back into patients, they can be targeted to attack tumors by co-administering HER2-specific mAbs, such as Herceptin (Figure 1C). Kudo and colleagues [75] designed a novel construct containing the high-affinity CD16 (FCGR3A) V158 variant with a CD8 hinge, transmembrane domains, along with signaling domains 4-1BB and CD3z (CD16V-BBz). When expressed on T cell surface, CD16V-BBz can bind antigen-specific mAbs with high affinity. Herceptin triggered CD16V-BBz mediated killing of HER2+ breast and gastric cancer cells; similar results were obtained with other antigen specific mAbs in various types of cancer models [75]. Furthermore, coadministration of CD16V-BBz T cells with immunotherapeutic mAbs exerted strong antitumor activity *in vivo* [75]. Thus, the therapeutic benefit and toxicity may be controllable by adjusting the amount of the infused targeting mAbs.

Similar constructs utilize anti-fluorescein isothiocyanate (FITC) scFv or avidin as extracellular domains were also reported [76, 77]. When expressed on the T cell surface, these T cells recognize various cancer types when bound with FITC-labeled or biotinylated mAbs (Figure 1C) resulting in T-cell activation, cytokine production and target lysis. These studies highlight an applicability of these novel CAR designs utilizing various mAbs currently in clinical use to treat patients with different types of cancers.

### Clinical application and toxicity

the first report of clinical use of HER2 CAR T cells is a case report of a serious adverse event following CAR T-cell treatment [16]. A HER2 positive (≥ 2+ as assessed by immunohistochemistry) colon cancer patient was treated with 3rd generation 4D5 CARs discussed above containing CD28, 4-1BB, and CD3ζ signaling moieties engineered T cells. However, this patient developed respiratory distress within 15 minutes of receiving a single dose of 10^10^ CAR T cells, followed by multiple cardiac arrests over the course of 5 days, eventually leading to death. Serum analysis four hours after treatment revealed marked increases in the cytokines IL-6, IFN-γ, GM-CSF, TNF-α, and IL-10. CAR T cells were found in the lung and abdominal and mediastinal lymph nodes, but not in tumor metastases. The immune-mediated recognition of TAAs in normal tissues is referred to as “on-target, off-tumor” toxicity. HER2 is not a tumor-specific antigen and is normally found in epithelial cells in the gastrointestinal, respiratory, reproductive, and urinary tracts and in skin, breast, and placenta, as well as in normal hematopoietic cells [78]. Therefore, the investigators attributed toxicity to the recognition of HER2 in lung epithelium by CAR T cells resulting in inflammatory cytokine release producing pulmonary toxicity and CRS causing multi-organ failure. In addition, it is likely that HER2(+) lung metastases of this patient could induce CRS mediated by antitumor effects of CAR T cells, which is referred to as “on-target efficacy” toxicity [79]. The autopsy results showed the highest levels of vector-containing cells were seen in the lung and abdominal/mediastinal lymph nodes, although these cells did not preferentially accumulate in metastatic deposits in the lungs and liver [16].

A recent clinical trial reported no significant toxicities of a separate second generation FRP5 HER2-specific CAR (4-1BB not incorporated) therapy for sarcoma [39]. Compared with above study at NCI, patients in this study were treated with lower maximum dose of HER2 CAR T cells without lymphodepleting chemotherapy and IL-2 support. Moreover, FRP5 CAR recognizes a discontinuous epitope within residues 11-169 of mature human HER2 protein facing away from the cell surface, while 4D5 CAR binds to the juxtamembrane region of HER2. The effective activation of CAR T cells can depend on the site of the CAR recognizing and binding epitope within the target antigen. Hence, FRP5-based CARs may be less likely than 4D5-based CARs to get activated by HER2 expressed at moderate levels by normal epithelial tissues. Although no significant toxicities were reported, neither was evidence of T cell expansion in the peripheral blood or IFN-γ release *in vivo* after CAR T cells infusion. One patient who received salvage chemotherapy in addition to CAR T cell infusion had an objective anti-tumor response. However, it is impossible to determine the relative contributions of the infused CAR T cells versus chemotherapy. Thus, a parallel comparison of FRP5 and 4D5-based HER2 CAR in a large group of patients would be required before a firm conclusion can be drawn.

In addition, investigators at Baylor College of Medicine have conducted different trials to determine the safety and antitumor efficacy of HER2-specific CAR T cells and CMV CTLs in patients with glioblastoma (GBM). In China, two institutes are evaluating HER2 CAR T cell-therapy in breast cancer and other solid tumors (Table 1).

**Table 1:** Clinical trials of HER2 CAR

Clinical Trials.gov Identifier	Phase	Cell source	CAR signaling domain	Disease	Sponsor	Status
NCT00902044	Phase 1	Patient autologous T cells	CD28-CD3ζ	Advanced sarcoma	Baylor College of Medicine, US	Recruiting participants
NCT01109095	Phase 1	CMV-specific CTLs	CD28-CD3ζ	GBM	Baylor College of Medicine, US	Active, not recruiting
NCT02442297	Phase 1	Patient autologous T cells	CD28-CD3ζ	GBM	Baylor College of Medicine, US	Recruiting participants
NCT00889954	Phase 1	TGFBeta resistant HER2 /EBV-CTLs	CD28-CD3ζ	Her2 (+) malignancy	Baylor College of Medicine, US	Active, not recruiting
NCT02547961	Phase 1 Phase 2	Patient autologous T cells	CD28-CD3ζ	Breast cancer	Fuda Cancer Hospital, Guangzhou, China	Recruiting participants
NCT01935843	Phase 1 Phase 2	Patient autologous T cells	4-1BB-CD3ζ	HER-2 (+) advanced solid tumors	Chinese PLA General Hospital, China	Recruiting participants

*Note: Data obtained from clinicaltrials.gov*.

### Combinatorial HER2 car T-Cell therapy

The microenvironment of solid tumors defends against an attack from the immune system and is the major barrier to treatment with immunotherapy [80, 81]. Systemic administration of antibodies that block the immunosuppressive checkpoints, such as cytotoxic T-lymphocyte associated protein 4 (CTLA-4) or programmed cell death protein-*1* (PD-1), resulted in improvement of outcomes for patients with solid tumors [82–84]. The CAR T cells can be inhibited by expressing PD-1 when it interacts with its ligands, PD-L1 and PD-L2 that are expressed on tumor cells [85]. John et al. [86] showed HER2 CAR T cells upregulated PD-1 following antigen stimulation *in vitro*, and blockade of PD-1 significantly enhanced the activation and proliferation of CAR T cells. In HER2 transgenic mice, they showed a significant enhanced tumor inhibition of two different HER2+ tumors treated with HER2 CAR T cells in combination with PD-1 blockade. Importantly, increased antitumor activities were not associated with side effects in normal tissue expressing the HER2 antigen. Similarly, armed oncolytic adenovirus expressing PD-L1 mini-body enhances the anti-tumor activity of HER2 CAR T cells in a HER2+ prostate cancer xenograft model [87]. In a more recent study, Beavis et al. [88]showed HER2 CAR T cells activation induces the expression of adenosine 2A receptor (A_2A_R), which in turn suppress CAR T cells. It is possible that A_2A_R blockade may enhance the efficacy of CAR T cells against tumors. Thus, combining HER2 CAR therapy with blocking inhibitory receptors offer the potential to enhance antitumor activity of CAR therapy.

Furthermore, effective combination immunotherapy for solid tumors could induce robust immune response and overcome the immunosuppressive tumor microenvironment. One potential approach of combining of HER2 CAR T cells with agonist antibodies targeting activation receptor 4-1BB (α-4-1BB) was recently developed [89]. Another approach, called adoptive cell transfer incorporating vaccination (ACTIV) therapy [90], utilizes dual-specific T cells expressing both a Her2-specifc CAR and a gp100 TCR in combination with recombinant vaccinia virus encoding human gp100 (VV-gp100) can eradicate large solid tumors in mice. A lymphodepleting preconditioning regimen and IL-2 has been shown to enhance antitumor effects of HER2 CAR T cells [91] was necessary for tumor eradication in this study. However, rigorous clinical trials are required to confirm the safety and efficacy of these combination therapies.

### Building safer HER2 cars

**Figure 2:**
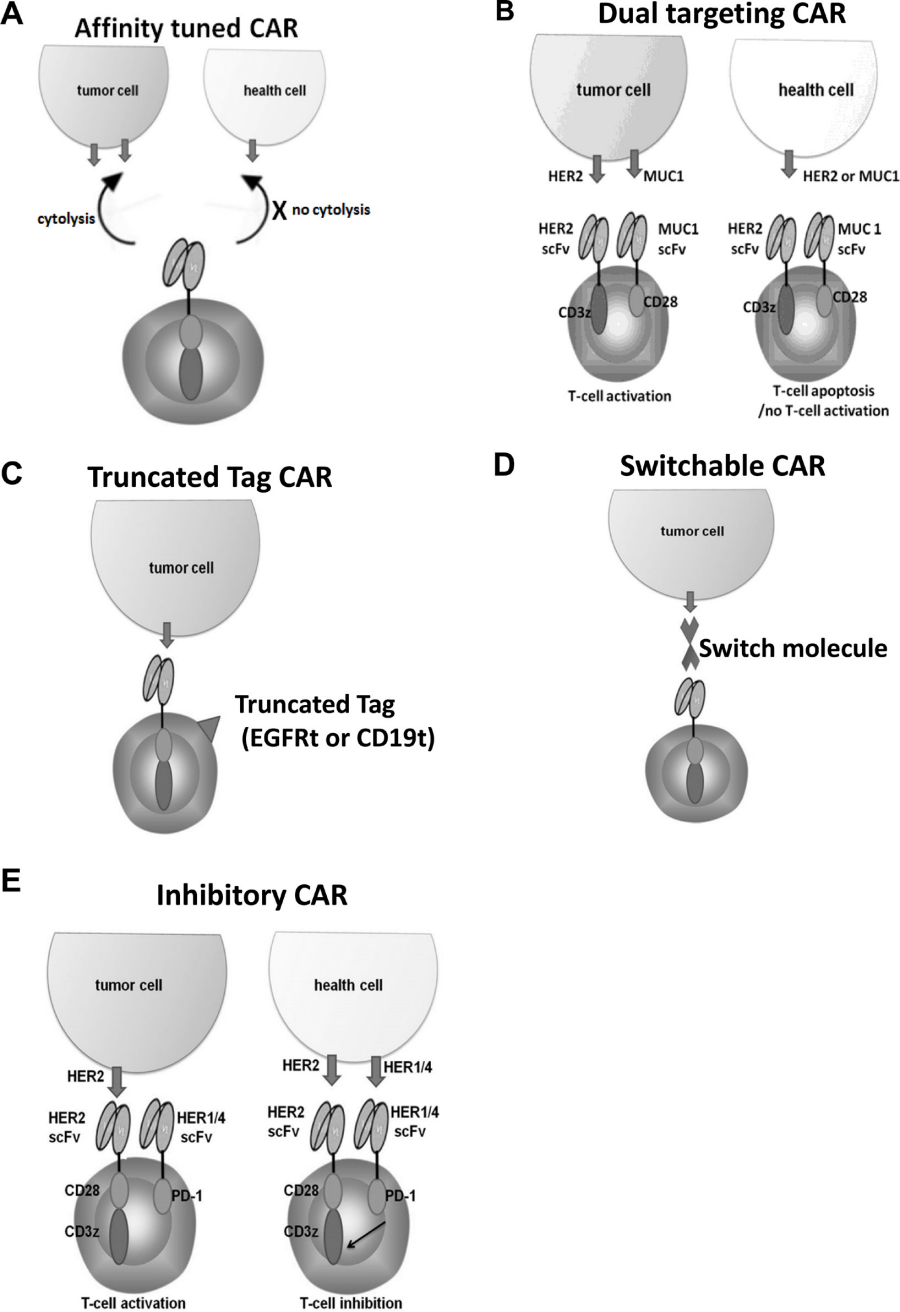
Strategies applied to improve safety of HER2 CAR therapy (**A**) Affinity-tuned HER2 CAR cells exhibit cytolytic activity against tumor cells but spare normal cells expressing physiologic target levels. (**B**) The full activation of “dual targeting CAR" requires both first generation CAR and a costimulated CAR directed against HER2 and MUC1 expressing tumor cells. HEE2 expressing health cells induce apoptosis of CAR T cells, while MUC1 expressing health cells do not activate CAR T cells. (**C**) CAR T cells are co-expressed with a truncated surface molecule tag. The administration of a mAb directed against the tag allows the elimination of the CAR cells. (**D**) FITC or PNE (GCN4) tagged anti-Her2 Fab switch molecules can redirect CAR-T cells (specific for the corresponding FITC or PNE) to HER2-expressing breast cancer cells. (**E**) Costimulated HER2 CAR T cells co-express a PD-1-based inhibitory CAR (ICAR) that recognizes other members of the HER family (HER1/4) which are co- expressed in healthy tissues with low level of HER2.

HER2 CAR T-cell therapies have life-threatening “on-target, off-tumor” toxicity [16]. To mitigate toxicity of CAR therapy, selecting scFvs that have proper target affinity for CAR constructs may lead to minimal normal tissue toxicity but retain antitumor activity. Liu et al. [92] showed high-affinity HER2 CAR T cells can recognize different levels of HER2 expressing cells, including normal cells with undetectable HER2 by flow cytometry. While affinity-tuned HER2 CAR T cells exhibited robust antitumor efficacy similar to high-affinity cells, they displayed significantly reduced reactivity against physiologic levels of HER2 (Figure 2A). Thus, the use of affinity-tuned scFvs offers a strategy to promote the wider use of CAR T cells against validated targets widely overexpressed in solid tumors.

In addition to focusing on selecting scFv with proper affinity for HER2 CAR T cell therapy, several novel design*s* for HER2 CAR constructs have been explored. First, various “dual targeting CARs” have been developed to improve safety [93–95]. A “dual targeting CAR” approach is to modify T cells expressing two different antigen specific-CARs; one CAR is first-generation CAR which contain the CD3z signaling domain for activation (signal 1), while another CAR with different specificity is modified second-generation CAR which contain the only CD28 and/or 4-1BB costimulatory signaling domain for costimulaiton (signal 2). This strategy would reproduce the physiological signal 1 and 2 checkpoints of T cell activation. Wilkie et al. [95] reported an attempt to implement this principle by exploring HER2/CD3-ζ for activation and MUC1/CD28 for costimulation (Figure 2B). This study demonstrated that “dual-targeting” CAR T cells kill HER2+ tumor cells efficiently and proliferate in a manner that requires co-expression of MUC1 and HER2 by tumor cells, coupled with diminished damage to normal tissues expressing single antigen. Second, including a depletion marker such as truncated EGFR or CD19 in the CAR construct to allow the T cells to be destroyed on exposure to a specific mAbs in the case of severe toxicity (Figure 2C) [96–98].

Moreover, incorporating an inducible caspase 9 suicide gene within CAR T cells discussed previously [24] would provide an additional safety control. However, similar to depletion marker strategy, these strategies result in irreversible loss of engineered T cells from circulation and do not solve the intrinsic lack of control associated with conventional CAR-T cell therapy. Novel platforms of switchable CAR T cells [99–102] in which the activity of the engineered cell is controlled by dosage of switch molecules are being developed. One of these studies applied this approach to HER2-expressing breast cancers by engineering switch molecules through site-specific incorporation of FITC or grafting of a peptide neo-epitope (PNE) into the anti-HER2 4D5 Ab fragment Fab (Figure 2D). Both switch formats can redirect CAR T cells (specific for the corresponding FITC or PNE) and also HER2-expressing breast cancer cells, which result in CAR T cells activation in dose dependent manner *in vitro* and eradiation of tumors in mouse xenograft models. This strategy may afford comparable efficacy with improved safety owing to switch based control of the CAR T-cell response.

Another novel design is to engineer costimulated HER2 CAR T cells to co-express a PD-1 or CTLA4-based inhibitory CAR (ICAR) that recognizes other members of the HER family (HER1/4) which are co expressed in healthy tissues with low HER2 (Figure 2E). Thus, ICAR and costimulated CAR signals will neutralize each other in healthy tissue, whereas costimulated HER2 CAR signals will dominate in the tumor microenvironment [103]. Consequently, it is predicted that the effector function of the transduced T cells will be restricted to tumor sites.

In addition, using T cells with temporary expression of a RNA CAR via electroporation may provide a safe platform [104]. Importantly, starting treatment with a very low dose of CAR T cells may prevent severe immediate toxicity. Thus, a more restricted dose-escalation scheme should be evaluated in clinical trials [105].

### Conclusions and perspectives

CAR T cells are "living drugs" with the capacity to proliferate, persist and provide sustained functional immunity. The basic science, preclinical and clinical work has and continues to make HER2 an increasingly important therapeutic target for CAR therapy. Combining HER2 CAR T cell therapy with checkpoint inhibitors may have synergistic effects in augmenting antitumor responses. With recent improvements in gene delivery of NK cells that have led to the enhancement of homing, *in vivo* persistence, cytolytic activity and safety, CAR modified NK cells or virus specific CTLs holds great promise for the development of effective treatments. A better understanding of the multiples barriers seen in solid tumors and a combination of various safety strategies will drive advances in HER2 CAR engineering and in clinical trial design to control and/or minimize potential “off tumor, on target” toxicity of HER2 CAR therapy. We look forward to introducing this transformative therapy to more cancer patients.

## Abbreviations

HER2: human epidermal growth factor receptor 2
scFv: single-chain variable fragment
CARs: Chimeric antigen receptors
ACT: Adoptive cellular therapy
TILs: Tumor-infiltrating lymphocytes
CTLs: cytotoxic T lymphocytes
NK: Natural killer
ARs: ankyrin repeats
mAb: Monoclonal Antibody
TAAs: Tumor-associated antigens
CTLA-4: cytotoxic T-lymphocyte associated protein 4
PD-1: programmed death-1
